# Basolateral Secretion from Caco-2 Cells Pretreated with Fecal Waters from Breast Cancer Patients Affects MCF7 Cell Viability

**DOI:** 10.3390/nu13010031

**Published:** 2020-12-24

**Authors:** Christine Bobin-Dubigeon, Jean-Marie Bard, Trang-Huyen Luu, Françoise Le Vacon, Hassan Nazih

**Affiliations:** 1 EA 2160—IUML FR3473 CNRS, Université de Nantes, 44035 Nantes, France; christine.bobin-dubgeon@univ-nantes.fr (C.B.-D.); tranglh269@gmail.com (T.-H.L.); el-hassane.nazih@univ-nantes.fr (H.N.); 2 Institut de Cancérologie de l’Ouest, 44805 Saint-Herblain, France; 3 Biofortis, Mérieux Nutrisciences, 44800 Saint-Herblain, France; francoise.le.vacon@mxns.com

**Keywords:** microbiota, breast cancer, gut, lipid metabolism

## Abstract

We hypothesized that the role of microbiota in breast cancer relates to its influence on gut lipid metabolism. This was tested in an in vitro model combining MCF-7 and Caco-2 cells. A total of 32 women newly diagnosed for breast cancer before any treatment and 28 healthy women provided their stools. Bacterial DNA was amplified by qPCR targeting 16s rRNA specific to Bacteroidetes and Firmicutes phyla, *Lactobacillales* sp., *Clostridium* cluster IV, *Faecalibacterium prausnitzii*, *Clostridium* cluster XIVa, *Roseburia intestinalis*, *Blautia* sp., *Lactonifactor longoviformis*, *Bifidobacterium* sp., Coriobacteriaceae, *Eggertella lenta*, *Escherichia,* and *Shigella*. Fecal waters (FW) were quantified for short chain fatty acids (SCFA). Caco-2 cells grown on filter inserts were incubated apically with 10% FW for 24 h, and *LXR*, apolipoproteins *AIV,* and *E* gene expression were estimated by real time (RT) qPCR. Then, MCF-7 cells were incubated with the whole basolateral medium for 24 h, and their viability was estimated by 3-(4,5-Dimethyl-2-thiazolyl)-2,5-diphenyltetrazolium bromide (MTT) test. Regression models were used to determine the correlation between MCF-7 viability and bacteria relative abundance, Caco-2 cells lipid metabolism gene expression and stool composition, as well as microbiota composition and short chain fatty acids. Logistic regression models established disease odds ratios (OR) for MCF-7 viability and Caco-2 gene expression. The OR of MCF-7 viability was 1.05 (1.01–1.10) (OR (5th–95th), *p* = 0.04), while that of *apo AIV* gene expression was 0.63 (0.39–1.01), *p* = 0.055). Viability correlated with % *Bifidobacterium* sp. (21.18 ± 7.66, *p* = 0.008) and valerate (−2.849 ± 1.048, *p* = 0.009) (β ± s.d.). This study suggests that microbiota interacts with intestine cell lipid metabolism. Since these metabolites can reach breast cells by systemic circulation, we hypothesized that they may influence cancer disease.

## 1. Introduction

Several works indicate that disrupting lipid rafts in various breast cancer cell models may be a key factor for breast cancer cell proliferation and apoptosis [1,2,3,4,5]. The LXR (Liver X receptor) pathway has been shown to be involved in this process through its influence on cholesterol trafficking. LXR activation may deprive breast cancer cells of cholesterol by stimulating its efflux through the expression of ABCG1 (ATP-binding cassette sub-family G member 1), a cell carrier of cholesterol. This results in a decrease in cellular cholesterol content and a subsequent inhibition of cell proliferation as well as a stimulation of apoptosis [6]. Apolipoproteins, such as apolipoprotein (apo) E [7], whose synthesis is under the control of LXR, and apo AIV [8,9] may be involved in cholesterol efflux from normal and breast cancer cells. Both apolipoproteins are cholesterol carriers which are involved in the process of cholesterol efflux from cells. In addition, apo E has been shown to be able to inhibit breast cancer cell proliferation, this effect being related to cholesterol efflux. All together, these mechanisms highlight the interest of favoring cholesterol efflux from breast cancer cells in order to decrease their viability [5,6,7]. On the other hand, we and others have suggested that microbiota is altered in patients with breast cancer [10,11,12] and differs according to the severity of the disease [13]. However, it is still not clear whether microbiota composition influences the occurrence or the progression of breast cancer, or if its alteration is a consequence of the disease. Our hypothesis is that various lipid metabolites produced in the gut by microbiota or by the intestinal cells under its influence may reach breast cells through systemic circulation. Under this circumstance, these metabolites may interfere with breast cancer cell behavior. To test this hypothesis, we evaluated the capability of fecal water (FW) isolated from stools obtained from breast cancer patients and controls to regulate lipid metabolism genes in an intestinal cell model, differentiated Caco-2 cells grown on semipermeable membranes. We also determined if the basolateral medium of these cells was able to differently influence the behavior of MCF-7, a model of breast cancer cells.

## 2. Materials and Methods

### 2.1. Patients and Controls

This study included 29 healthy women and 32 patients with early stage breast cancer referred to our hospital (ICO René Gauducheau, Saint-Herblain, France). Our biobank has been declared to and authorized by the French Research Ministry (Declaration Number: DC-2018-3321). This declaration includes approval by a research ethics committee (CPPComité de Protection des Personnes) [14]. Informed consent was obtained from both the experimental and the control groups, granting permission to use their biological specimens and clinical-pathological data for research purposes, as required by the French legislation and the French committee for the protection of human rights. Use of any medication known to have an impact on microbiota was considered as an exclusion criterion (probiotics, antibiotics, or gastrointestinal drugs). The patient population was included in our earlier research [13]. Briefly, most patients were diagnosed for an invasive carcinoma of no specific type (76%), clinical stage 0 or 1 (80%), with a good prognostic grade (84% of grades I and II). All patients were positive for estrogen and progesterone receptor and negative for HER2. The tumor localization was mainly unifocal, with a median size of 12.5 mm. No carriers of tumor BRCA1 and or BRCA2 mutation were identified in the patient group.

Feces (*n* = 32) from newly diagnosed breast cancer patients before any therapy and control feces (*n* = 29) were freshly collected and immediately processed for fecal water and for the quantification of total and selected bacteria.

### 2.2. Materials

Dulbecco’s Modified Eagle’s Medium (DMEM, Cat#D5796), fetal bovine serum (FBS, Cat#F0804), glutamine (Cat#G7513), penicillin-streptomycin (Cat#PO781), HEPES buffer (Cat#HO887), non-essential amino acids (NEAA, Cat#M7145), fatty acid-free bovine serum albumin (BSA, Cat#A8806), 3-(4,5-Dimethyl-2-thiazolyl)-2,5-diphenyltetrazolium bromide (MTT, Cat#M5655), dimethyl sulfoxide (DMSO, Cat#D8418), primers for qPCR, and other chemicals that are not specifically indicated were from Sigma Aldrich (Lyon, France). TRIzol reagent (Cat#15596018) for RNA isolation was from Invitrogen (Cergy Pontoise, France). iSript^TM^ Reverse Transcription Supermix (Cat#170-8841) for real time (RT)-qPCR and iQ^TM^ SYBR Green Supermix (Cat#170-8886) were purchased from Bio-Rad (Marnes-la-Coquette, France). Transwells with 0.4 µm Pore Polyester Membrane Insert (Cat#665641) were from Greiner Bio-one^®^ (Courtaboeuf, France).

### 2.3. Quantification of Bacteria

All aliquots of fresh feces (2 g) were prepared and used for the bacterial DNA extraction, as previously described [13]. Real time quantitative PCR (RT qPCR) was used to determine the copy numbers of total bacteria and of some selected bacterial populations targeting 16s rRNA specific to Bacteroidetes and Firmicutes phyla, *Lactobacillales* sp., *Clostridium* cluster IV, *Faecalibacterium prausnitzii*, *Clostridium* cluster XIVa, *Roseburia intestinalis*, *Blautia* sp., *Lactonifactor longoviformis*, *Bifidobacterium* sp., Coriobacteriaceae, *Eggertella lenta*, *Escherichia,* and *Shigella* [9]. The number of bacteria for each bacterial population was expressed as log_10_ equivalent bacteria (noted log_10_ eq. bact.) per gram of fresh stool and expressed as percent of the total.

### 2.4. Preparation of Fecal Water (FW) Samples

After collection from breast cancer patients (*n* = 32) and control women (*n* = 29), 2 g (±0.1 g) of fresh stool were weighed in ultracentrifuge tubes. After adding 2 mL of sterile phosphate buffered saline (PBS) buffer, the tubes were well vortexed to obtain a homogeneous suspension. The tubes were then centrifuged at 77,000 g/min at 20 °C for 2 h using the Beckman Coulter Optima™ XPN ultracentrifuge. After the ultracentrifugation, the supernatant was filtered through a 0.22 µm filter. Finally, the supernatant was stored at −20 °C for future analyses.

### 2.5. Assay of Short Chain Fatty Acids in FW Samples

The short chain fatty acid (SCFA) assay method was adapted from the protocol described by Gourbeyre P et al. [15]. After centrifugation of the thawed FW samples (10,000× *g* for 15 min), the supernatants (30 μL) were added with 270 μL of 0.5 M oxalic acid. The SCFAs were analyzed by capillary gas-liquid chromatography method (SGE BP21 on a capillary column: 25 × 0.53 mm with a film thickness of 0.25 μm and nitrogen as the carrier gas: 20 mL/min). The injector and the detector temperatures were maintained at 270 °C and 250 °C, respectively; the oven temperature was 100 °C. The sample (1 μL) was introduced by injection without division with a flow rate of 50 mL/min, starting 1 min after injection. The concentration of SCFAs in FW samples was determined by comparison with an SCFAs solution of known concentration, analyzed under the same chromatographic conditions.

### 2.6. Cell Culture

The human adenocarcinoma breast cancer MCF-7 and Caco-2 cell lines were from European Collection of Animal Cell Cultures (ECACC, Salisbury, UK). MCF-7 cells were cultured in flasks in DMEM supplemented with 10% FBS, 1% glutamine, and 1% penicillin-streptomycin. DMEM medium supplemented with 10% FCS, 1% HEPES, 1% NEAA, 1% glutamine, and 1% penicillin-streptomycin was used for Caco-2 cells. The cells were maintained in a humidified incubator at 37 °C in atmosphere of 5% CO_2_.

### 2.7. Preparation of the Differentiated Caco-2 Monolayer

Caco-2 cells were seeded at a density of 2.5 × 10^5^ cells/well on Transwell with 0.4 µm Pore Polyester Membrane Insert. The inserts were placed in 12-well plates, allowing access to the apical and the basolateral sides of the cell monolayers. The apical and the basolateral mediums were replaced three times a week. The experiments were carried out 21 days after seeding. Caco-2 cells at this stage were differentiated into enterocytes.

### 2.8. Incubation of the Caco-2 Monolayer with FW Samples

The FW samples diluted to 1/10th in culture medium containing 0.1% BSA were added on the apical side of Caco-2 cells. The negative control was Caco-2 cells incubated with the culture medium 0.1% BSA. The transepithelial electrical resistance (TEER) measurement of the Caco-2 monolayers was carried out at 0 h and 24 h of incubation using the Millicell Electrical Resistance System (Millipore) to verify the integrity of the monolayer. TEER measurement method was based on Ohm’s Law method described by Srinivasan [16]. After 24 h of incubation, the mediums from apical and basolateral compartments were collected and stored at −20 °C for analysis. The mRNA was extracted from Caco-2 cells prior to the analysis of gene expression using RT-qPCR, as described below.

### 2.9. MCF-7 Cell Viability Assay

MCF-7 cells were seeded in 96-well plates at a density of 10^4^ cells/well in 200 μL of culture medium and left to adhere overnight. The seeding medium was then removed, and cells were treated with basolateral medium from Caco-2 cells incubated with FW samples. The percentage of cell viability was determined after 24 h of incubation. For MTT assay, MTT solution (50 µL of 2.5 mg/mL) was added to each well to get formazan crystals. After 4 h of incubation, the liquid in the well was removed, and formazan was solubilized in 200 µL of DMSO. The absorbance of formazan solution at 570 nm was measured using SpectraMax 190. Cell viability was then calculated as a percentage of the control that was treated with basolateral medium from Caco-2 cells which were not incubated with FW.

### 2.10. Gene Expression Analysis by RT-PCR

Total RNA of Caco-2 cells was isolated using TriZol Reagent, and then cDNA was prepared by using iScript^TM^ Reverse Transcription Supermix, according to the manufacturer’s instructions. An initial priming step for 5 min at 25 °C was followed by reverse transcription phase of 30 min at 42 °C and completed by RT inactivation step of 5 min at 85 °C. Quantitative PCR was performed on a MyiQ2 Real-Time PCR Detection System (Bio-Rad) using iQ^TM^ SYBR Green Supermix. PCR was carried out for 45 cycles of 95 °C for 30 s and 60 °C for 30 s. *LXR, ApoE,* and *ApoA-IV* relative expressions were standardized to the reference gene *18S* expression using the ΔΔCT method. The sequences of the primers used are shown in Table 1.

## 3. Results

The characteristics of the patient population were extensively described in our previous paper [13]. Briefly, most breast cancer patients were diagnosed for ductal invasive carcinoma (76%) at clinical stage 0 or 1 (80%) with a good prognostic grade (84% of grades I and II), 100% being RH+ and HER2−. The tumor localization was mainly unifocal with a median size of 12.5 mm. No carriers of tumor *BRCA1* and or *BRCA2* mutation were identified in the patient group.

To examine the integrity of the Caco-2 monolayers before and after they were incubated with FW, we used the quantitative TEER technique. After 24 h of incubation with FW, the value of TEER of Caco-2 monolayers used in our assay varied between 180 and 300 Ω·cm^2^. There was no difference between TEER values obtained after 24 h incubation of FW from patients and controls (235.5 Ω·cm^2^ and 243.6 Ω·cm^2^, respectively, *p* = 0.21).

### 3.1. Characteristics of the Studied Population and MCF-7 Cell Viability

Table 2 shows that patients and controls did not significantly differ for age, menopausal status, or BMI.

As shown in Table 3, the MCF-7 cell viability after 24 h exposure to the basolateral medium derived from differentiated Caco-2 cells pre-incubated with FW differed significantly between patients and controls. Table 3 also shows that lipid metabolism gene expression in Caco-2 cells pre-incubated with FW from patients and controls did not differ significantly, although a tendency towards a lower expression of the *Apo AIV* gene in patients than in controls was observed (*p* = 0.055).

### 3.2. MCF-7 Viability and Bacteria Relative Abundance

As shown in Table 4, the viability of MCF-7 cells after 24 h incubation with the basolateral medium of Caco-2 cells previously exposed to FW from the whole studied population was positively correlated with the relative abundance of *Bifidobacterium* sp. in the crude stools. A negative correlation was also observed between MCF-7 cell viability and the presence of valerate in the FW. The other SCFAs were not related with MCF-7 viability. There was no significant relationship between MCF-7 cell viability and the expression of lipid metabolism genes in Caco-2 cells. Among the parameters correlated with MCF-7 cell viability in univariate analysis, only *Bifidobacterium* sp. and valerate in FW remained significantly correlated in the multiple regression analysis.

### 3.3. Caco-2 Cells Lipid Metabolism Gene Expression and Stool Composition

Table 5 indicates that the expression of *Apo AIV* gene in Caco-2 cells exposed to FW was positively correlated with the relative abundance of the crude stools in *Bacteroidetes* and the FW concentration in acetate, propionate, and butyrate. Valerate was not significantly correlated with *Apo AIV* gene expression. In the multiple regression model, only *Bacteroidetes* remained significantly correlated with *Apo AIV* gene expression, while there remained a tendency towards a positive correlation of *Apo AIV* gene with propionate.

The correlations between *LXR* gene expression in Caco-2 cells exposed to FW and the concentration of SCFA in FW or the relative abundance of bacterial genes in the initial stools are given in Table 6. *LXR* gene expression was negatively correlated with *Blautia* sp. and all SCFA: acetate, propionate, butyrate, and valerate. In the multiple regression analysis, only the correlation with *Blautia* sp. remained significant.

### 3.4. Relationship between Stool Microbiota and Short Chain Fatty Acids in Fecal Water

Table 7 shows the correlations obtained between stool microbiota composition and the presence of SCFA in FW. Only the presence of *Coriobacteriacae* was significantly and negatively correlated with acetate (*p* = 0.04), propionate (*p* = 0.007) and, to a lesser extent, butyrate (*p* = 0.09) content in FW. A trend towards a negative correlation was also observed between *Lactonifactor longoviformis* and acetate content (*p* = 0.06).

## 4. Discussion

This *in vitro* study was run to test the hypothesis that various lipid metabolites produced in the gut by microbiota or by the intestinal cells under its influence may reach breast cells through systemic circulation, and that these metabolites may interfere with breast cancer cell behavior. The main result of this study is that the basolateral medium of Caco-2 cells has a different effect on MCF-7 cell viability when pre-incubated with FW obtained from patients or controls. This suggests that this medium contains some soluble metabolites able to influence the breast cancer cell behavior. These metabolites may represent a direct production of bacteria crossing the cell membranes or the gut cell metabolites synthesized under the influence of bacterial metabolites. To rule out any artefact result, which could be due to an alteration of Caco-2 cell permeability, during the experiments, we measured the TEER value. This value is widely used to examine the integrity of cellular barriers before assessment of drug or chemical transport. According to Srinivasan B et al. [16], the Caco-2 monolayer that generates a TEER of 150–400 Ω·cm^2^ restricts the diffusion of substances across the barrier. Thus, Caco-2 TEER values in the range of 180–300 Ω·cm^2^ in the present study allowed us to confirm the barrier integrity of the cell monolayers in the presence of fecal waters from patients and controls.

SCFAs are well known metabolites produced by microbiota. They have been suggested to influence cancer progression. Although there is a large body of evidence on the influence of SCFA on cancers affecting the digestive tract, results on breast cancer are rather scarce. *In vitro*, butyrate was shown to be able to induce apoptosis of MCF-7 cells [17]. Butyrate and propionate were able to inhibit MCF-7 cell proliferation, while butyrate seemed to be more potent than propionate [18]. Butyrate and valerate were also shown to be able to inhibit MCF-7 cell proliferation and to induce differentiation by a mechanism involving remodeling of intracellular Ca^2+^ homeostasis [19]. In patients, baseline formate and acetate plasma levels were recently identified as potential predictive markers to select patients who will achieve clinical benefit from gemcitabine carboplatine therapy and to identify those who should not be treated [20]. Our results indicate that valerate present in FW incubated at the apical pole of Caco-2 cells was significantly and independently related to a lower capability of the basolateral medium of Caco-2 cells to induce MCF-7 cell proliferation. This would suggest a major influence of this short chain fatty acid. However, our study did not allow us to determine if this was a direct effect of this fatty acid which would cross the enterocyte membrane or if its effect would involve cell products secreted in the basolateral medium. The direct effect of this short chain fatty acid has been tested on several cancer lines, including breast cancer cells. Indeed, significant anti-proliferative effects with a positive dose-dependent relationship were observed in all tested cell lines [21]. Additionally, encapsulated nanoparticles containing valerate were injected via the tail vein into xenograft mouse models of liver tumors to test their effectiveness. In this study, the authors showed that the systemic delivery of these nanoparticles containing valerate demonstrated a reduction of tumor cell proliferation and had a significantly improved survival rate in the studied mouse models. Histone deacetylase (HDAC)-inhibiting functions of valerate were also revealed in this study [21]. HDAC inhibitors, such as SCFAs, are considered as pro-apoptotic agents. No data exist on the effect of valerate on apoptosis of MCF-7 cells. However, cell death has been detected in liver cancer cells after incubation with valerate. In this study, the pro-apoptotic pathway Bak-1 and Caspase3 was reported to be the main mechanism of action of valerate [21]. In addition to this pathway, plasma membrane Ca^2+^-ATPase (PMCA) proteins are known to be involved in cell proliferation and apoptosis. PMCA proteins are detected in normal fully differentiated breast epithelial tissues and are essential in the Ca^2+^ homeostasis and possibly other signaling pathways. Valerate induced upregulation of PMCA4b proteins in MCF-7 cells, and this correlates with their differentiation and apoptosis [19]. Valerate has also been shown to enhance PMCA4b isoform protein expression and differentiation in gastric and colon cancer cells [22]. Further study using FW or SCFAs isolated from FW with the procedure described by Dobrowolska-Iwanek J. et al. [23] directly in contact with MCF7 cells could be helpful to clarify this point. Paying particular attention to the extraction method for SCFA would be necessary in this context to avoid any loss of some metabolites with low water solubility, as suggested by Dobrowolska-Iwanek J. et al. [23].

The MCF-7 cell viability was significantly and positively related to the percent of *Bifidobacterium* sp. in the FW incubated with Caco-2 cells. This seems to be in good agreement with our previous results obtained in patients newly diagnosed for breast cancer, indicating that the presence of *Bifidobacterium* sp. in stools is related to tumor size and grade [13]. This relationship was independent in the multivariate model. Therefore, it can be ruled out that the effect of this bacterial strain is explained by its capability to produce SCFA.

One of our hypotheses was that FW from patients with breast cancer would differ from controls in their capability to induce genes involved in lipid metabolism in Caco-2 cells. As a matter of fact, our previous results obtained in patients showed that the expression of LXR dependent genes in tumor is related to breast tumor characteristics [24]. In addition, our previous *in vitro* studies clearly showed that cholesterol carriers, which are under the control of LXR, are able to reduce MCF-7 cell proliferation [6,7]. Here, we did not show any relationship between the expression of *LXR* or *Apo E* genes in Caco-2 cells after incubation with FW. However, a clear trend towards a negative relationship was observed for *Apo AIV* gene expression. Looking forward to an influence of SCFA or some bacteria in stools on *Apo AIV* gene expression, we found that this gene was positively related to acetate, butyrate, and propionate, as well as to the percent of *Bacteroidetes*. This confirms our previous observation that butyrate stimulates the production of Apo AIV in Caco-2 cells [9]. However, in the multiple regression analysis, only the percent of *Bacteroidetes* remained significantly related to *Apo AIV* gene expression, suggesting that the relationship with SCFA depends on the bacterial composition of stools. Nevertheless, our previous results in patients did not show any relationship between the percent of *Bacteroidetes* and the severity of the disease, while the absolute number of *Bacteroidetes* was positively associated with the stage of the tumor [13]. Despite the fact that our results did not show any relationship between *LXR* gene expression in Caco-2 cells and the disease, considering our previous results on LXR and breast cancer, we decided to analyze if this expression was influenced by the concentration of short chain fatty acids or the presence of some bacteria in FW. All fatty acids studied were negatively associated with *LXR* gene expression as well as the percent of *Blautia* sp. In the multiple regression analysis, only the percent of *Blautia* sp. remained significantly and negatively associated with *LXR* gene expression. In our previous results obtained in newly diagnosed patients, *Blautia* sp. was clearly related with the severity of the disease [13]. Therefore, combined with our *in vitro* results, which indicate that LXR driven genes may be protective against the disease [6,7,24], these results suggest that *Blautia* sp. could influence tumor behavior through its negative effect on LXR.

It would have been expected from the knowledge on the influence of microbiota on SCFA production that the FW content in these SCFA would correlate with the presence of some bacteria in stool. The only significant correlation was found between some SCFA, basically acetate and propionate and *Coriobacteriacae*. However, the presence of these bacteria was not found to be related to MCF7 cell survival in our model. Therefore, the relationships that we observed between valerate and MCF7 cell survival may reflect the influence of some other diet component or bacteria which were not quantified in our study.

It may be suggested from this preliminary study that some metabolites produced in stools under the influence of microbiota may interfere with both lipid metabolism in intestine and breast cancer tumor behavior. This study suffers from some weaknesses. In particular, we did not record diet within the days preceding stool collection. Therefore, we cannot exclude any interaction between food components and our results. Further clinical studies are needed to identify to which extent the gut may play a role in breast cancer.

## 5. Conclusions

Stool microbiota interacts with intestine cell lipid metabolism and influences breast cancer tumor behavior. This suggests a role of gut in breast cancer, which should be evaluated in further clinical studies.

## Figures and Tables

**Table 1:** Sequence of the primers used in this study.

Gene Name	Sequences (5′-3′)	
*18S*	F- GATGCGGCGGCGTTATTCC	R- CTCCTGGTGGTGCCCTTCC
*LXR*	F- GCTCCCACCGCTGCTCTC	R- TGCCCTTCTCAGTCTGTTCCAC
*ApoE*	F- CTGCGTTGCTGGTCACATTCC	R- CGCTCTGCCACTCGGTCTG
*ApoA-IV*	F- CAACTCAATGCCCTCTTC	R- CTCCTCCTTCAGTTTCTCC

**Table 2:** Main characteristics of the studied population.

	Controls ** (*n* = 29)	Breast Cancer ** (*n* = 32)	Odds Ratio ***
Age (years)	53.0 (47.0–62.0)	61.0 (50.5–69.0)	1.05 (1.00–1.10)
			*p* = 0.06
BMI (Kg/m^2^)	23.9 (22.5–25.6)	23.4 (21.5–26.0)	1.00 (0.87–1.16)
			*p* = 0.95
Menopausal status (yes/no/%yes)	19/10/65.5%	23/9/71.9%	
		*p* = 0.59 *	

(*) Chi Square. (**) Median (25th–75th). (***) Odds ratio (5th–95th).

**Table 3:** MCF-7 cell viability after incubation with the basolateral medium of Caco-2 cells exposed to fecal waters from patients and controls and lipid metabolism gene expression in the intestine model.

	Controls * (*n* = 29)	Breast Cancer * (*n* = 32)	Odds Ratio **
*LXR*	1.29 (0.97–1.68)	1.07 (0.82–1.42)	0.68 (0.26–1.77)
			*p* = 0.43
*Apo E*	1.18 (0.85–1.33)	0.94 (0.71–1.27)	0.88 (0.38–2.03)
			*p* = 0.77
*Apo AIV*	3.03 (2.00–3.92)	2.20 (1.67–3.31)	0.63 (0.39–1.01)
			*p* = 0.055
MCF7 viability 24 h	90.40 (87.31–95.46)	97.54 (87.93–106.99)	**1.05 (1.01–1.10)**
			***p* = 0.04**

(*) Median (25th—75th). (**) Odds ratio (5th—95th). Significant results in bold characters.

**Table 4:** Relationship between abundance of given bacterial genes in stools, short chain fatty acids (SCFAs) in fecal water (FW) or Caco-2 cell lipid metabolism gene expression and MCF-7 cell viability after incubation with the basolateral medium of Caco-2 cells exposed to FW from the whole population.

	Univariate Model	Mutlivariate Model
	β ± s.d.	β ± s.d.
Total bacteria	0.112 ± 9.117 (*p* = 0.99)	
% *Bacteroidetes*	−0.306 ± 0.214 (*p* = 0.16)	
% *Firmicutes* Phyllum	0.154 ± 0.089 (*p* = 0.09)	
% *Bifidobacterium* sp.	**21.18 ± 7.66 (*p* = 0.008)**	**20.57 ± 7.27 (*p* = 0.006)**
% *Lactobacillales*	−89.02 ± 73.41 (*p* = 0.23)	
% *Escherischia/Shigella*	11.50 ± 30.51 (*p* = 0.71)	
% *C. Leptum* Cluster IV	0.266 ± 0.148 (*p* = 0.077)	
% *Clostridium* Cluster XIVa	0.075 ± 0.134 (*p* = 0.58)	
% *F. prausnitzii*	0.193 ± 0.204 (*p* = 0.35)	
% *Roseburia intestinalis*	−0.237 ± 1.194 (*p* = 0.84)	
% *Blautia* sp.	2.124 ± 1.850 (*p* = 0.26)	
% *Eggerthella Lenta*	48.78 ± 45.28 (*p* = 0.29)	
% *Coriobacteriacae*	2.33 ± 2.19 (*p* = 0.29)	
% *Lactonifactor longoviformis*	−255.23 ± 223.39 (*p* = 0.26)	
Acetate	0.092 ± 0.062 (*p* = 0.15)	
Propionate	0.295 ± 0.186 (*p* = 0.12)	
Butyrate	0.278 ± 0.156 (*p* = 0.08)	
Valerate	**−2.849 ± 1.048 (*p* = 0.009)**	**−2.765 ± 0.991 (*p* = 0.007)**
*LXR*	−1.084 ± 3.135 (*p* = 0.73)	
*Apo E*	0.624 ± 2.753 (*p* = 0.82)	
*Apo AIV*	−1.83 ± 1.324 (*p* = 0.17)	

Significant results in bold characters.

**Table 5:** Relationship between *apo AIV* gene expression in Caco-2 cells exposed to FW from the whole studied population and short chain fatty acids in FW or abundance of given bacterial genes in stools.

	Univariate Model	Multivariate Model
	β ± s.d.	β ± s.d.
Total bacteria	−0.93 ± 0.87 (*p* = 0.29)	
% *Bacteroidetes*	**0.06 ± 0.02 (*p* = 0.004)**	**0.063 ± 0.017 (*p* = 0.0006)**
% *Firmicutes* Phyllum	0.005 ± 0.009 (*p* = 0.63)	
% *Bifidobacterium* sp.	−0.16 ± 0.79 (*p* = 0.84)	
% *Lactobacillales*	5.57 ± 7.16 (*p* = 0.44)	
% *Escherischia/Shigella*	−0.92 ± 2.95 (*p* = 0.76)	
% *C. Leptum* Cluster IV	−0.003 ± 0.01 (*p* = 0.81)	
% *Clostridium* Cluster XIVa	0.017 ± 0.013 (*p* = 0.19)	
% *F. prausnitzii*	0.008 ± 0.020 (*p* = 0.70)	
% *Roseburia intestinalis*	0.069 ± 0.115 (*p* = 0.55)	
% *Blautia sp.*	0.025 ± 0.18 (*p* = 0.89)	
% *Eggerthella Lenta*	2.44 ± 4.41 (*p* = 0.58)	
% *Coriobacteriacae*	−0.188 ± 0.21 (*p* = 0.38)	
% *Lactonifactor longoviformis*	−10.78 ± 21.82 (*p* = 0.62)	
Acetate	**0.015 ± 0.0059 (*p* = 0.01)**	−0.003 ± 0.009 (*p* = 0.74)
Propionate	**0.063 ± 0.016 (*p* = 0.0003)**	0.057 ± 0.03 (*p* = 0.056)
Butyrate	**0.050 ± 0.014 (*p* = 0.0007)**	0.027 ± 0.025 (*p* = 0.29)
Valerate	0.121 ± 0.106 (*p* = 0.26)	−0.092 ± 0.107 (*p* = 0.40)

Significant results in bold characters.

**Table 6:** Relationship between *LXR* gene expression in Caco-2 cells exposed to FW from the whole studied population and short chain fatty acids in FW or abundance of given bacterial genes in stools.

	Univariate Model	Multivariate Model
	β ± s.d.	β ± s.d.
Total bacteria	−0.117 ± 0.378 (*p* = 0.76)	
% *Bacteroidetes*	0.002 ± 0.009 (*p* = 0.82)	
% *Firmicutes* Phyllum	−0.007 ± 0.004 (*p* = 0.06)	
% *Bifidobacterium* sp.	−0.566 ± 0.329 (*p* = 0.09)	
% *Lactobacillales*	3.027 ± 3.058 (*p* = 0.33)	
% *Escherischia/Shigella*	−0.778 ± 1.263 (*p* = 0.54)	
% *C. Leptum* Cluster IV	−0.006 ± 0.006 (*p* = 0.35)	
% *Clostridium* Cluster XIVa	0.006 ± 0.006 (*p* = 0.29)	
% *F. prausnitzii*	−0.013 ± 0.008 (*p* = 0.13)	
% *Roseburia intestinalis*	−0.022 ± 0.049 (*p* = 0.66)	
% *Blautia* sp.	**−0.172 ± 0.074 (*p* = 0.02)**	**−0.184 ± 0.072 (*p* = 0.01)**
% *Eggerthella Lenta*	−1.235 ± 1.889 (*p* = 0.52)	
% *Coriobacteriacae*	−0.015 ± 0.092 (*p* = 0.87)	
% *Lactonifactor longoviformis*	−8.738 ± 9.299 (*p* = 0.35)	
Acetate	**−0.0056 ± 0.0025 (*p* = 0.03)**	0.0005 ± 0.0045 (*p* = 0.91)
Propionate	**−0.016 ± 0.007 (*p* = 0.04)**	0.0019 ± 0.014 (*p* = 0.89)
Butyrate	**−0.017 ± 0.006 (*p* = 0.009)**	−0.0178 ± 0.012 (*p* = 0.14)
Valerate	**−0.089 ± 0.045 (*p* = 0.05)**	−0.0359 ± 0.052 (*p* = 0.49)

Significant results in bold characters. FW: fecal water.

**Table 7:** Correlation between stool microbiota composition and short chain fatty acids in FW.

	Actetate	Propionate	Butyrate	Valerate
	β ± s.d.	β ± s.d.	β ± s.d.	β ± s.d.
Total bacteria	19.39 ± 18.36 (*p* = 0.30)	6.36 ± 6.19 (*p* = 0.31)	11.76 ± 7.23 (*p* = 0.11)	−0.20 ± 1.07 (*p* = 0.85)
% *Bacteroidetes*	−0.40 ± 0.44 (*p* = 0.36)	−0.08 ± 0.15 (*p* = 0.61)	−0.09 ± 0.18 (*p* = 0.60)	−0.02 ± 0.03 (*p* = 0.47)
% *Firmicutes* Phyllum	0.06 ± 0.19 (*p* = 0.74)	−0.03 ± 0.06 (*p* = 0.59)	−0.02 ± 0.07 (*p* = 0.78)	0.01 ± 0.01 (*p* = 0.46)
% *Bifidobacterium* sp.	8.04 ± 16.52 (*p* = 0.63)	−4.00 ± 5.55 (*p* = 0.47)	4.10 ± 6.58 (*p* = 0.54)	−0.22 ± 0.95 (*p* = 0.82)
% *Lactobacillales*	−67.68 ± 150.81 (*p* = 0.65)	−49.78 ± 50.48 (*p* = 0.33)	−1.89 ± 60.27 (*p* = 0.97)	−2.20 ± 8.70 (*p* = 0.80)
% *Escherischia/Shigella*	39.77 ± 61.87 (*p* = 0.52)	9.58 ± 20.88 (*p* = 0.65)	−7.96 ± 24.75 (*p* = 0.75)	1.47 ± 3.57 (*p* = 0.68)
% *C. Leptum* Cluster IV	0.32 ± 0.31 (*p* = 0.30)	0.05 ± 0.10 (*p* = 0.63)	0.02 ± 0.12 (*p* = 0.85)	0.02 ± 0.02 (*p* = 0.25)
% *Clostridium cluster XIVa*	0.34 ± 0.27 (*p* = 0.22)	0.06 ± 0.09 (*p* = 0.53)	0.03 ± 0.11 (*p* = 0.78)	0.01 ± 0.02 (*p* = 0.55)
% *F. prausnitzii*	0.28 ± 0.42 (*p* = 0.50)	0.09 ± 0.14 (*p* = 0.52)	0.08 ± 0.17 (*p* = 0.63)	0.02 ± 0.02 (*p* = 0.35)
% *Roseburia intestinalis*	2.50 ± 2.40 (*p* = 0.30)	−0.11 ± 0.82 (*p* = 0.89)	0.09 ± 0.97 (*p* = 0.93)	−0.01 ± 0.14 (*p* = 0.97)
% *Blautia* sp.	−0.88 ± 3.80 (*p* = 0.82)	−0.82 ± 1.28 (*p* = 0.47)	−0.85 ± 1.51 (*p* = 0.58)	0.05 ± 0.22 (*p* = 0.83)
% *Eggerthella Lenta*	−104.39 ± 91.94 (*p* = 0.26)	−18.75 ± 31.22 (*p* = 0.55)	−25.28 ± 36.93 (*p* = 0.50)	1.95 ± 5.35 (*p* = 0.72)
% *Coriobacteriacae*	**−9.27 ± 4.32 (*p* = 0.04)**	**−3.98 ± 1.42 (*p* = 0.007)**	−2.98 ± 1.75 (*p* = 0.09)	−0.06 ± 0.26 (*p* = 0.80)
% *Lactonifactor Longoviformis*	−869.45 ± 444.87 (*p* = 0.06)	−104.17 ± 154.07 (*p* = 0.50)	−232.65 ± 180.61 (*p* = 0.20)	−26.05 ± 26.23 (*p* = 0.32)

Significant results in bold characters.

## Data Availability

The data presented in this study are available on request to the corresponding author. The data are not publicly available due to ethical reason.

## Abbreviations

Apo	Apolipoprotein
BRCA1	Breast cancer 1
BRCA2	Breats cancer 2
BSA	Bovine serum albumin
CT	Cycle threshold
DMEM	Dulbecco’s Modified Eagle’s Medium
DMSO	Dimethyl sulfoxide
FBS	Fetal bovine serum
FW	Fecal water
HEPES	[**4**-(2-Hydroxyethyl)-1-piperazinyl]-ethanesulfonic acid
HER2	Human Epidermal Growth Factor Receptor-2
LXR	Liver X Receptor
MTT	3-(4,5-Dimethyl-2-thiazolyl)-2,5-diphenyltetrazolium bromide
NEAA	Non essential amino acid
PBS	Phosphate buffered saline
qPCR	Quantitative polymerase chain reaction
RT	Reverse transcriptase
RT qPCR	Real time quantitative polymerase chain reaction
RT-qPCR	Reverse transcription quantitative polymerase chain reaction
SCFA	Short chain fatty acid
TEER	Transepithelial electrical resistance
